# Measurement of Thickness at the Inferior Border of the Mandible Using Computed Tomography Images: A Retrospective Study including 300 Japanese Cases

**DOI:** 10.3390/tomography9040098

**Published:** 2023-06-22

**Authors:** Nobuhiro Ueda, Miki Zaizen, Yuichiro Imai, Tadaaki Kirita

**Affiliations:** 1 Department of Oral and Maxillofacial Surgery, Nara Medical University, 840 Shijo-cho, Kashihara, Nara 634-8522, Japan; 2 Department of Oral and Maxillofacial Surgery, Rakuwakai Otowa Hospital, 2 Chinji-cho, Yamashima-ku, Kyoto 607-8062, Japan

**Keywords:** head and neck cancer, mandibular reconstruction, mandibular thickness, medication-related osteonecrosis of the jaw, oral cancer, osteoradionecrosis

## Abstract

Vascularised fibular free flaps are integral to reconstructive surgery for head and neck tumours. We investigated the morphological characteristics of the mandible to improve the incidence of plate-related complications after surgery. Using standard radiological software, thickness measurements of the inferior or posterior margin of the mandible were obtained from computed tomography images of 300 patients at seven sites: (1) mandibular symphysis, (2) midpoint between the mandibular symphysis and mental foramen, (3) mental foramen, (4) midpoint between the mental foramen and antegonial notch, (5) antegonial notch, (6) mandibular angular apex (gonion), and (7) neck lateral border of the dentate cartilage. Relationships between age, sex, height, weight, the number of remaining teeth in the mandible, and the thickness of each mandible were also investigated. Measurement point 1 had the largest median mandibular thickness (11.2 mm), and measurement point 6 had the smallest (5.4 mm). Females had thinner measurements than males at all points, with significant differences at points 1, 2, 3, 4, and 7 (*p* < 0.001). Age and number of remaining teeth in the mandible did not correlate with mandibular thickness; however, height and weight correlated at all points except point 6. Thickness measurements obtained at the sites provide a practical reference for mandibular reconstruction. Choosing the fixation method based on the measured thickness of the mandible at each site allows for sound plating.

## 1. Introduction

Segmental mandibulectomy is frequently selected for surgical management of malignant or benign tumours of the mandible, resulting in mandibular defects [1,2]. Therefore, mandible reconstruction is often performed to restore the impaired functional and aesthetic appearance. The vascularised free fibular flap (FFF) technique remains the gold standard in this regard [3]. In recent years, with an increase in cases of osteoradionecrosis (ORN) and medication-related osteonecrosis of the jaw (MRONJ) [4,5], similar FFFs have been widely used [6,7].

Bone fixation in FFF-based mandibular reconstruction relies primarily on the use of miniplates or reconstruction plates that provide stability to the osteotomy junction. Recently, with the development of computer-aided surgical simulation (CASS) and the clinical application of computer-aided design and manufacturing solutions, patient-specific implants (PSI) have been used [8,9]. With these technological developments, aesthetic reconstructive results have been achieved. In addition, efforts to restore occlusal function by placing dental implants in the reconstructed bone have received considerable attention.

However, in cases of malignant tumours, ORN, and MRONJ, bone and soft tissue healing conditions are often unfavourable. Therefore, plate-related complications such as plate extrusion, plate fracture, screw loosening, malunion, or non-union often occur [10,11,12,13,14]. Among these, the frequency of malunion with inadequate bone union is relatively high, ranging from 5 to 45.9%. 

Stresses are concentrated proximally in the mandible due to occlusal forces. In particular, high stress occurs in the mandibular angle and mandibular neck, which may explain the tendency for malunion in these regions [15]. When malunion occurs, the plate fracture risk due to occlusal forces increases, and prosthetic reconstruction cannot be performed without additional surgery for remobilization. 

Malunions have been studied regarding surgical precision and the type of plate to be fixed. However, this problem continues, and long-term stability has not been achieved. Although CASS has become widespread, the morphology of the bone itself for bone regeneration at the junction of the mandible and the reconstructed bone has not been studied. The anatomical morphology of the mandible and the fibula is somewhat well understood. However, there are few reports of fine measurements regarding normal anatomy that are suitable for use in bone junction analyses, and they are insufficient for adding to CASS. 

As part of an anatomical study using computed tomography (CT), the thickness of the area around the inferior margin of the mandible (i.e., the area to be bone-jointed during mandibular reconstruction) was measured in the current study. The data from this study can be used as a reference for mandibular reconstruction, mandibular fracture repair, diagnostic imaging, and anatomical studies. Furthermore, CASS, which considers the morphology and thickness of the mandible, may reduce the incidence of plate-related complications.

## 2. Materials and Methods

### 2.1. Patients

Patients who underwent head and neck CT examinations at the Department of Oral and Maxillofacial Surgery, Nara Medical University Hospital, between 2017 and 2021 were included in the study. Specific diseases included tumours, cysts, and inflammation in the oral and maxillofacial soft tissues and maxilla. Patients whose disease was in the mandible or whose mandibular measurements were affected by metal artefacts were excluded. Patient characteristics and images were extracted from the electronic medical records.

### 2.2. Variables

**Figure 1:**
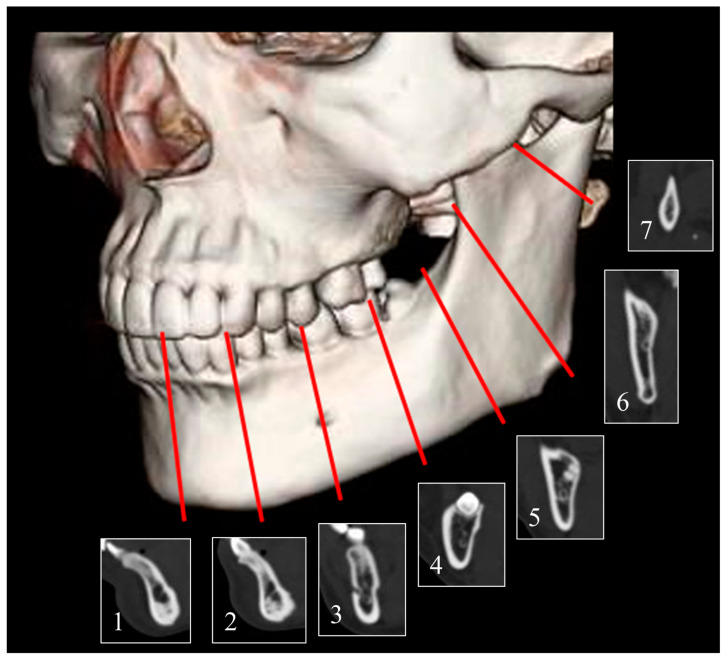
Measurement points of the inferior border of the mandible. (1) Mandibular symphysis; (2) midpoint between the mandibular symphysis and mental foramen; (3) mental foramen; (4) midpoint between the mental foramen and antegonial notch; (5) antegonial notch; (6) mandibular angular apex (gonion); and (7) neck lateral border of the dentate cartilage.

The variables studied were age, sex, height, weight, and the number of remaining teeth in the mandible. The CT examinations were performed using a SOMATOM Definition AS (Siemens Healthcare, Erlangen, Germany). The acquisition protocol included the following imaging parameters: tube voltage of 120 kV, tube current of 100 mA, tube rotation time of 0.50 s, pitch of 0.8, slice thickness of 0.625 mm, matrix of 512 × 512, and DFOV of 150 mm. Images were analysed using radiograph display software (AquariusNET Viewer; Ter-aRecon, Durham, NC, USA) to measure the thickness of the mandible on one side. Typically, in mandibular reconstruction, a titanium fixation plate is placed at the stress concentration site, defined as the compression zone of the mandible due to occlusion, and then fixed with bicortical screws. In this study, the mandibular thickness was measured at the following points: 5–7 mm above the inferior margin of the mandible, where screws are clinically placed for such load-bearing fixation: (1) mandibular symphysis; (2) midpoint between the mandibular symphysis and mental foramen; (3) mental foramen; (4) midpoint between the mental foramen and antegonial notch; (5) antegonial notch; (6) mandibular angular apex (gonion); and (7) neck lateral border of the dentate cartilage (Figure 1).

Measurements with CT images were performed according to a method that has already been proven to be highly reproducible in previous studies [16]. First, oral and maxillofacial clinicians with 3 years of CT imaging experience worked with an experienced specialist certified by the Japanese Society of Oral and Maxillofacial Surgeons with 17 years of CT imaging experience. Both reviewers were examined and calibrated using 30 sample images. The intraclass correlation coefficients (ICC) for both reviewers were ICC (2,1) = 0.906, 95% CI = 0.622 < ICC < 0.966, indicating that the measurements were reliable. The clinician obtained one final set of measurements. Even after calibration, the aforementioned specialist supervised the entire image review process. Both reviewers were blinded to detailed patient information.

**Figure 2:**
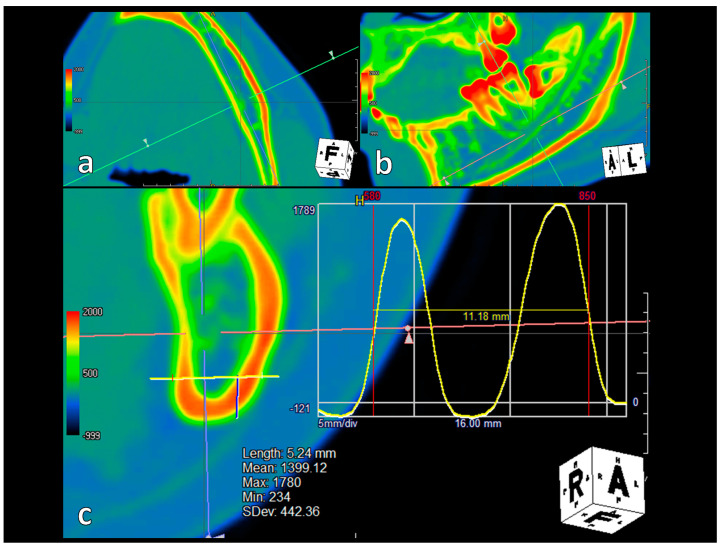
Measurement method at measurement point 4. (**a**) Customised axial: Adjust the axis to appear perpendicular to the buccal cortical bone. (**b**) Customised sagittal: Adjust the axis to appear horizontal to the mandible’s inferior border. (**c**) Customised coronal: Measurements are taken with a digital ruler 5–7 mm above the inferior margin of the mandible (blue line), specifying the profile curve as in the path of the bicortical screw placement (yellow line). The full width at half maximum is used to measure the buccolingual thickness of the mandible.

Particular attention was paid to measuring the thickness of the mandible at the point where the bicortical screw is normally placed (along the buccal cortex at the inferior or posterior margin of the mandible). At each of the above points, a cross-section of the mandible was displayed in axial, coronal, and sagittal images in software, and a profile curve was specified with a digital ruler at a position 5–7 mm above the inferior margin of the mandible, perpendicular to the buccal cortex as in the path of bicortical screw placement. The full width at half maximum of the resulting profile curve was used to measure the width of the buccolingual thickness of the mandible, buccal cortex bone, trabecular bone, and lingual cortex bone, respectively (Figure 2).

### 2.3. Ethical Approval

The institutional review board (IRB) of Nara Medical University Hospital approved this study (approval number 3348). Owing to the study’s retrospective nature, the patient consent requirement was waived. Therefore, we published the study plan on the hospital’s website homepage to guarantee an opt-out opportunity, as directed by the IRB.

### 2.4. Statistical Analyses and Validation

The Mann–Whitney U test was used to compare the relationship between mandibular thickness and sex. Pearson’s product-moment correlation coefficient was used to compare the relationship among mandibular thickness, height, and weight, for which a normal distribution was confirmed. Spearman’s rank correlation coefficient was used to determine the relationship between mandibular thickness and age and the number of remaining teeth in the mandible, for which a normal distribution was not found. All statistical analyses were performed using EZR (Saitama Medical Centre, Jichi Medical University, Saitama, Japan), a graphical user interface for R (R Foundation for Statistical Computing, Vienna, Austria). Two-tailed *p*-values < 0.05 were considered statistically significant throughout.

## 3. Results

**Figure 3:**
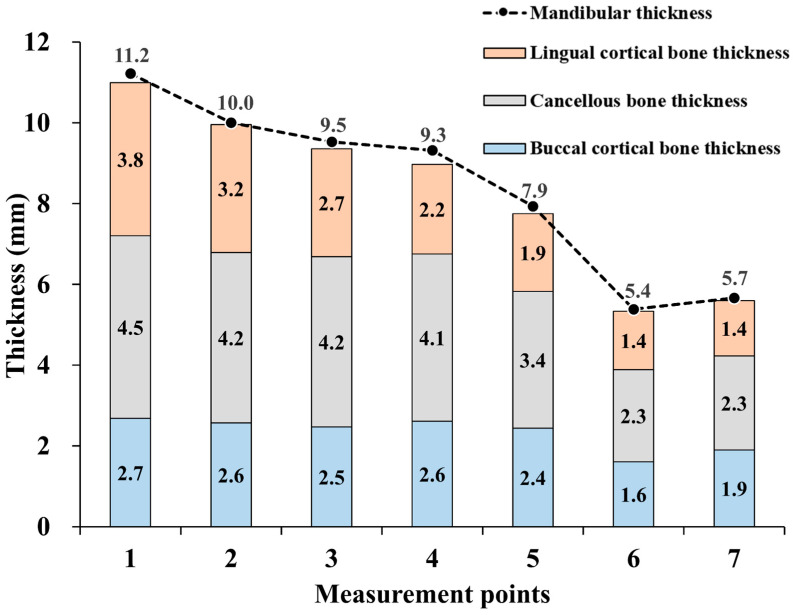
The thickness of the mandible at each measurement point. Median thickness of the mandible plus its components: buccal, trabecular, and lingual cortical bone.

Mandibular thickness was measured from CT images of 135 males and 165 females. Table 1 lists the characteristics of the 300 participants, and Figure 3 presents the median mandibular thicknesses at the seven measurement points. Measurement point 1 (mandibular symphysis) had the thickest median mandibular thickness at 11.2 mm, and point 6 (mandibular angular apex) had the thinnest, with a median thickness of 5.4 mm. The mandibular body was thicker than the mandibular angle and ramus.

Regarding cortical bone thickness, the lingual cortical bone was thicker than the buccal cortical bone at points 1 and 2. At point 3, the two were comparable, whereas the buccal cortical bone was thicker than the lingual cortical bone at points 4, 5, 6, and 7.

**Figure 4:**
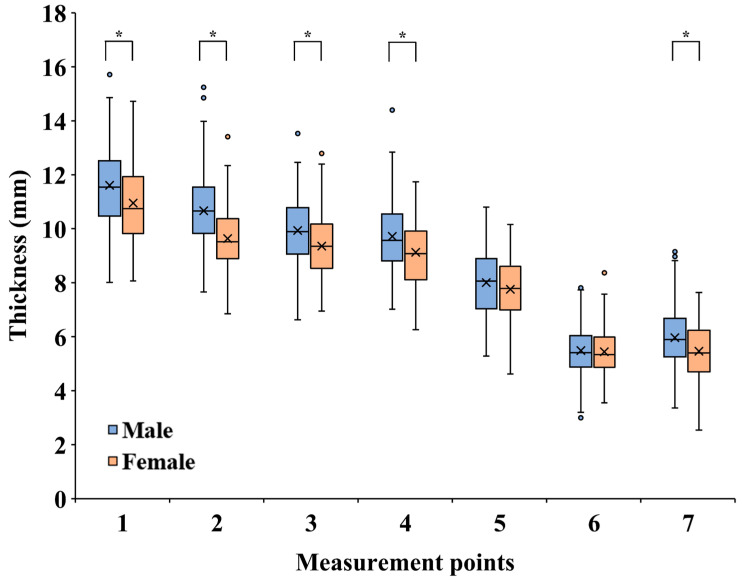
Data on mandibular thickness for males and females at each measurement point. * *p* < 0.001.

Regarding the relationship between mandibular thickness and sex (Figure 4), females had a smaller mandibular thickness than males at all measurement points. Points 1, 2, 3, 4, and 7 showed significant differences (*p* < 0.001). Age and the number of remaining teeth in the mandible did not correlate with mandibular thickness. However, height and weight correlated with mandibular thickness at all points except point 6 (Table 2).

## 4. Discussion

CT or cone-beam CT (CBCT) mandibular measurements are common and have been used in orthodontic [17], temporomandibular joint [18], dental implant [19,20], oropharyngeal airway [21], inferior alveolar nerve [22,23], mandibular third molar [24], and coronoid foramina [25] studies. However, these were limited to the alveolar region [20,24], inferior alveolar nerve region [19,22,23], mandibular branch [17,25,26], and mandibular condyle [18]. The thickness of the mandible near the inferior margin is partly understood based on clinical experience, imaging studies [27], and anatomy [28]; however, such data are rarely reported in the literature. Furthermore, data on middle-aged and older patients who may be candidates for reconstructive surgery are lacking, as few reports are limited to data on mandibular fractures in younger patients. In CASS, which is based on CT images, extensive data from these images is required to understand whether the area designated as the junction of the mandible and fibula is thicker or thinner than the common mandible. In this study, we retrospectively studied 300 adult Japanese individuals of various ages, focusing on mandibular thickness near the inferior margin, which is important for the fixation of mandibular reconstruction.

The mandible was thickest at measurement point 1 (mandibular symphysis) and thinnest at points 6 and 7 (angle of the mandible to the mandibular ramus). This trend was consistent with a report on 150 young dentate adults in Maryland, USA [27]. Compared to previous reports, mandibular body measurements tended to be 0.5–1 mm thinner, which may be due to ethnic factors. The median thickness from the angle of the mandible to the mandibular ramus was similar, ranging from 5–6 mm. As there are no reports on the measurement of the buccal, trabecular, and lingual cortical bones using CT, we measured each of them in this study. The buccal cortical bone was thicker in the anterior region of the mandible at points 1 and 2 (distal to the mandible). In contrast, the buccal cortical bone was approximately the same thickness at point 3. The buccal cortical bone was thicker in the anterior mandible at points 1 and 2, while it was generally similar at point 3. The trend of thicker lingual cortical bones across all points was similar to that observed in an analysis of frozen human heads in Texas, USA [28].

Significant differences were found between the mandibular thickness and sex at most measurement points. A previous report [27] showed a similar trend, with males having thicker mandibles, although the difference was insignificant. In the mandibular alveolar region, a correlation between bone thickness, age, and the number of remaining teeth in the mandible has been reported [29,30]. Contrary to our expectations, the present study revealed that mandibular thickness did not change with age or the number of remaining teeth in the mandible in the mandibular inferior region. Most measurement points correlate with mandibular thickness in relation to height and weight. Since no studies have compared mandibular thickness to sex, age, the number of remaining teeth in the mandible, height, or weight, these data would be helpful during mandibular reconstruction.

Many experienced oral surgeons are likely to have acquired knowledge of the mandibular thickness and fixation methods required during reconstruction in their clinical practise. Despite this, the frequency of plate-related complications remains high. Malunions, non-unions, plate fractures, and screw loosening are often the result of choosing a fixation method that cannot withstand biomechanical stress. Finite Element Analysis has rapidly gained popularity over the past decade, with many reports on PSI used for mandibular reconstruction [31,32,33]. PSIs have been devised in various shapes, and all of them exhibit reliable fixation stability compared to conventional plate systems. On the other hand, primary stability against expected masticatory forces is believed to be adequate for both miniplates, reconstruction plates, and PSI [34]. Despite such in vitro analysis, observational studies have shown a higher rate of incomplete bone fusion in the PSI group [11,35,36]. 

Unlike mandibular fracture surgery and mandibular osteotomy, which preserve the periosteum, segmental mandibulectomy involves the loss of the periosteum on the resected side. The periosteum of the preserved mandibular area is also detached to place the plate. Postoperatively, surgical site infections are common, and radiation therapy may be added. In short, it is a very difficult environment for bone regeneration. However, to the best of our knowledge, there is no previous report that established a relationship between mandibular inferior border thickness and plate-related complications. Additionally, the methods currently used do not consider the morphological characteristics of the mandible, which remains a glaring shortcoming. Fixation during mandibular reconstruction requires investigating fixation methods that can adequately withstand the stresses received. As there are differences in mandibular thickness based on sex, height, and weight, there may be differences in the concept of fixation based on each of these factors. Fixation of the mandibular branch is particularly important as it is a known site of stress concentration and susceptibility to plate-related complications, and the thinness of the mandible should be considered.

One limitation of this study is that the data represent measurements obtained from CT images, not cadavers or live gross specimens. However, no alternative techniques are available for measuring the thickness of healthy mandibles, and the number of cadaveric specimens is limited. Regarding the study population, we have not been able to identify patients with osteoporosis. In these patients, there is a potential for bias in the measurement of mandibular cortical bone thickness. In terms of imaging measurements, CT has thicker imaging slices and lower resolution than CBCT. However, for clinical applications in mandibular malignancies, ORN, and MRONJ, the analysis in this study was performed using CT, which is commonly used to diagnose these diseases. More to the point, analysis with CT used in clinical practise was necessary to add this knowledge to the CASS.

## 5. Conclusions

The thickness measurements obtained in this study provide a practical reference for mandibular reconstruction. Further, selecting a fixation method based on mandibular thickness measurements at each site is possible. In addition, if this knowledge can be added to the CASS, it will lead to sound plating that is stable in the long term.

## Figures and Tables

**Table 1:** Clinical characteristics of 300 patients.

Factors	Category	*N* = 300
Sex	Male	135
	Female	165
Age (years)	20–39	60
	40–59	77
	60–79	125
	≥80	38
Number of remaining teeth in the mandible	<5	14
	5–9	34
	10–13	96
	≥14	156
Height (cm)	<150	22
	150–159	126
	160–169	94
	170–179	50
	≥180	8
Body weight (kg)	<50	69
	50–59	86
	60–69	89
	70–79	40
	≥80	16

***N*** (Number).

**Table 2:** Correlations between mandibular thickness and each factor.

Measurement Points	Age ^a^		Number of Remaining Teeth ^a^		Height ^b^		Body Weight ^b^	
	R	*p* Value	R	*p* Value	R	*p* Value	R	*p* Value
1	0.011	0.86	0.102	0.078	0.235 *	<0.001 **	0.125	0.030 **
2	–0.018	0.76	0.109	0.059	0.392 *	<0.001 **	0.274 *	<0.001 **
3	0.060	0.30	0.065	0.26	0.300 *	<0.001 **	0.171	0.003 **
4	0.113	0.051	0.005	0.93	0.226 *	<0.001 **	0.122	0.035 **
5	–0.102	0.078	0.053	0.36	0.133	0.021 **	0.137	0.018 **
6	0.034	0.56	−0.045	0.44	0.028	0.63	0.046	0.42
7	0.051	0.38	0.043	0.46	0.148	0.010 **	0.153	0.008 **

* Correlation. ** Statistically significant. ^a^ Spearman’s rank correlation coefficient. ^b^ Pearson’s product-moment correlation coefficient.

## Data Availability

The data presented in this study are available upon request from the corresponding authors.
